# Regulation of Translation of ATF4 mRNA: A Focus on Translation Initiation Factors and RNA-Binding Proteins

**DOI:** 10.3390/cells15020188

**Published:** 2026-01-20

**Authors:** Pauline Adjibade, Rachid Mazroui

**Affiliations:** Oncology Unit, Department of Molecular Biology, Medical Biochemistry and Pathology, Faculty of Medicine, Laval University, Québec, QC G1V 0A6, Canada

**Keywords:** ATF4 mRNA, translation regulation, integrated stress response, uORFs, eIFs, RNA demethylases, RNA-binding proteins, stress granules

## Abstract

Cells are continuously exposed to physiological and environmental stressors that disrupt homeostasis, triggering adaptive mechanisms such as the integrated stress response (ISR). A central feature of ISR is the selective translation of activating transcription factor 4 (ATF4), which orchestrates gene programs essential for metabolic adaptation and survival. Stress-induced acute ATF4 expression occurs in diverse mammalian cell types and is typically protective; however, chronic activation contributes to pathologies including cancer and neurodegeneration. Canonical ISR (c-ISR) is initiated by phosphorylation of eIF2α in response to stressors such as endoplasmic reticulum or mitochondrial dysfunction, hypoxia, nutrient deprivation, and infections. This modification suppresses global protein synthesis while promoting ATF4 translation through upstream open reading frames (uORFs) in its 5′UTR. Recently, an alternative pathway, split ISR (s-ISR), enabling ATF4 translation independently of eIF2α phosphorylation, was identified in mice, suggesting ISR adaptability, though its relevance in humans remains unclear. Under normal conditions, cap-dependent translation predominates, mediated by the eIF4F complex and requiring the activity of eIF2B at its initial steps. During translational stress, eIF2α phosphorylation inhibits eIF2B activity, resulting in the formation of stalled initiation complexes, which can aggregate into stress granules (SGs). SGs sequester mRNAs and translation initiation factors, further repressing global translation, while ATF4 mRNA largely escapes sequestration, enabling selective translation. This partitioning highlights a finely tuned regulatory mechanism balancing ATF4 expression during stress. Recent advances reveal that, beyond cis-regulatory uORFs, trans-acting factors such as translation initiation factors and associated RNA-binding proteins critically influence ATF4 translation. Understanding these mechanisms provides insight into ISR plasticity and its implications for development, aging, and disease.

## 1. Introduction

Cells constantly face internal and external physiological and environmental stressors that threaten homeostasis. To adapt, they activate protective stress response mechanisms, including the integrated stress response (ISR). A main component of the ISR is the activation of a conserved translational control pathway that upregulates the cAMP-responsive transcription factor ATF4, a key regulator of the integrated stress response (ISR) [1,2,3,4,5]. Once induced, ATF4 activates one of two gene expression programs, depending on its level and duration of expression: high levels or prolonged expression promote expression of pro-apoptotic proteins such as C/EBP homologous protein (CHOP [6]), while moderate levels or acute expression activate stress-adaptive proteins, including antioxidants and proteins with roles in amino acid synthesis essential for metabolic adaptation and survival [7,8]. Thus, the regulation of ATF4 mRNA translation is a critical stress-induced pathway that affects either cell death or survival. In mammals, stress-induced ATF4 translation occurs in various cell types, such as stem cells, neurons, myocytes, blood cells, and cardiomyocytes, and alteration of its regulation can contribute to diseases including cancer and neurodegeneration [9,10,11,12].

ATF4 translation is induced primarily during the canonical ISR (c-ISR), triggered by diverse stressors: chemical inducers of mitochondrial [13,14] or endoplasmic reticulum (ER) stress [15,16], hypoxia [17,18,19], amino acid [20,21] or glucose [22,23] deprivation, anticancer drugs and RNA-based therapies [24,25,26], and physiological processes like embryonic development, hematopoiesis, and senescence [27,28,29]. It is also activated in pathological conditions, including infections [30,31,32].

Stress-induced ATF4 involves phosphorylation of eIF2α [2,33,34,35], a key translational regulator [36]. This modification suppresses global cap-dependent translation while selectively promoting ATF4 translation, enabling stress-specific gene expression [37]. Among these, GADD34, ATF5, and CHOP encode for mRNAs that are translationally regulated by uORFs, potentially involving similar ATF4 translational regulatory mechanisms [1,2,3,4,5]. Recently, an alternative stress-like pathway, the split ISR (s-ISR), was identified in mice, allowing ATF4 translation independently of eIF2α phosphorylation, suggesting ISR adaptability [38]. Whether s-ISR contributes to aberrant ATF4 activation in human cells under non-stress conditions remains unclear. In this review, “translational stress” refers to cellular states that are generated by c-ISR, marked by ATF4 induction alongside global translational repression, typically via eIF2α phosphorylation.

## 2. Brief Review of Translation and Its Regulation During Translational Stress

**Figure 1:**
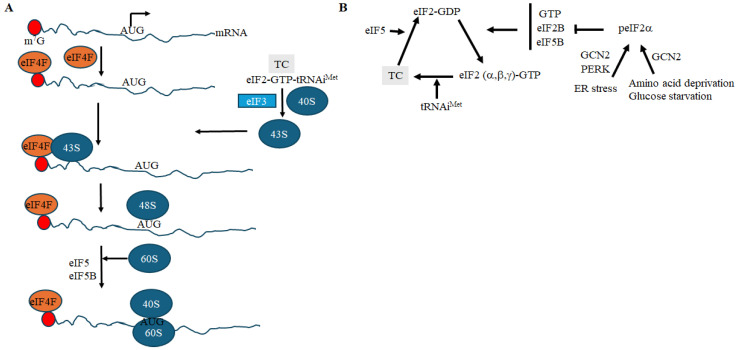
(**A**) A simplified view of the different steps of the cap-dependent translation initiation. The initiation of translation begins with recognition of the 5′ m^7^GTP cap (m^7^G; red circle) structure by the eIF4F complex. eIF4F then recruits the 43S pre-initiation complex (PIC), composed of the eIF3, 40S ribosomes, the eIF2-GTP-Met-tRNAᵢ^Met^ ternary complex (TC), and other initiation factors. This 43S PIC scans the 5′UTR of eIF4F-bound mRNAs to locate the start codon, AUG. Recognition of the start codon triggers conformational changes in the 43S PIC, resulting in the formation of the 48S complex. The 60S ribosomal subunit joins to form the 80S ribosome, which is competent for translation. (**B**) Regulation of the formation of TC and its recycling. Binding of eIF2-GTP to Met-tRNAᵢ^Met^ forms a TC complex. Hydrolysis of GTP of the TC, stimulated by eIF5, leads to the release of eIF2-GDP. Displacement of eIF2-GDP from Met-tRNAi is enhanced by eIF5B. Recycling of eIF2-GDP to its GTP-bound form and reassembly of the TC is mediated by eIF2B. Translational stress (e.g., ER stress, amino acids, and glucose deprivation) activates stress kinases (e.g., GCN2 and PERK) that phosphorylate eIF2α. peIF2α blocks eIF2B activity and thus prevents exchange of eIF2-GDP to eIF2-GTP, impairing TC assembly. In (**B**), arrowheads denote activation and blunt-ended lines indicate inhibition.

The majority of cellular mRNAs are translated through canonical cap-dependent translation, which begins with recognition of the 5′ m^7^GTP cap structure of the mRNA by the eIF4F complex (Figure 1A). This complex comprises the canonical cap-binding protein eIF4E, the RNA helicase eIF4A, which unwinds secondary structures in the 5′ untranslated region (UTR), and the scaffold protein eIF4G1 [39,40,41,42]. eIF4F recruits eIF3, which is associated with the 43S pre-initiation complex (PIC), composed of the 40S ribosomal subunit, the eIF2-GTP-Met-tRNAᵢ^Met^ ternary complex (TC), and other initiation factors such as eIF1 and eIF1A (Figure 1A). The 43S PIC scans the 5′UTR of eIF4F-bound mRNAs in the 5′ to 3′ direction and locates the start codon, typically the first AUG within a favorable Kozak context. Recognition of the start codon triggers conformational changes in the 43S PIC, resulting in the formation of the 48S complex. At this point, GTP hydrolysis by the γ-subunit of eIF2, stimulated by the GTPase-activating protein eIF5, leads to the release of eIF2-GDP. Recycling of eIF2-GDP to its GTP-bound form and reassembly of the TC is facilitated by eIF2B, the guanine nucleotide exchange factor (GEF) (Figure 1B) [43,44]. Finally, the 60S ribosomal subunit joins the 48S complex to form the 80S ribosome, which is competent for translation elongation [40] (Figure 1A). This canonical-cap dependent translation does not generally allow ATF4 translation under normal growth conditions, while its downregulation upon translational stress enables ATF4 translation.

**Figure 2:**
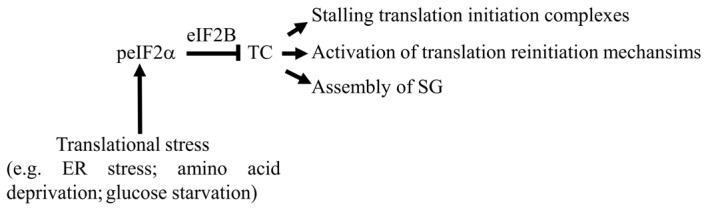
Consequences of TC downregulation upon stress. The loss of TC can induce formation of translation initiation complexes that are translationally deficient. Accumulation of those inactive initiation complexes can trigger the assembly of SGs, which may contribute to the inhibition of general translation. Downregulation of TC activates translation of a specific class of uORF-containing mRNAs via reinitiation mechanisms. Arrowheads denote activation, and blunt-ended lines indicate inhibition.

Specific eIF2 kinases, PKR, PERK, GCN2, and HRI, phosphorylate eIF2α in response to various stress signals that activate translational stress [33,45]. Phosphorylated eIF2α (peIF2α) acts as a competitive inhibitor of eIF2B, blocking GEF activity and thus preventing GDP–GTP exchange (Figure 2). This impairs TC formation, with multiple consequences on translation. Reduction in TC levels can either prevent the formation of 48S complexes or allow formation of translation initiation complexes that are stalled in an inactive form, as they lack functional TC. Accumulation of these inactive initiation complexes can trigger the formation of stress granules (SGs). These phase-dense cytoplasmic condensates, composed of small ribosomal subunits, mRNA, eIFs, and RNA-binding proteins (RBPs), assemble during translational stress [46], further contributing to the inhibition of general translation initiation by sequestering mRNAs away from the ribosomes that remain active during stress [47,48,49,50]. Consistent with this, we [25] and others [48,51] have shown that a subset of ATF4 mRNA localizes to SGs under ER stress in a translationally repressed state. However, under these conditions, the majority of ATF4 mRNA escapes SG sequestration and instead is found associated with translating ribosomes. This partitioning may underscore a finely tuned regulatory mechanism controlling whether ATF4 mRNA is silenced or translated during stress. Consequences of TC downregulation are summarized in Figure 2.

The activation of ATF4 translation is closely linked to the unique features of its 5′UTR [52]. Foundational studies identified the critical role of upstream open reading frames (uORFs) within the 5′UTR of ATF4 mRNA in mediating its stress-dependent translation initiation [6,10,16,18,53]. Advances in RNA translation technologies such as ribosome profiling (ribo-seq), which maps ribosome-protected fragments across the transcriptome [54], and quantitative translation initiation sequencing (QTI-seq), which pinpoints translation initiation sites [55], along with translational reporter assays have significantly expanded our understanding of uORF-mediated regulation of ATF4.

While much attention has been directed toward the cis-regulatory features of the 5′UTR, the role of trans-acting factors, including translation initiation factors (eIFs) and translational regulators, in regulating ATF4 translation has only recently begun to emerge. This review discusses recent discoveries on the implication of trans-acting factors in ATF4 translation during translational stress and beyond.

## 3. Regulation of ATF4 Translation by Its 5′UTR: An Overview

**Figure 3:**
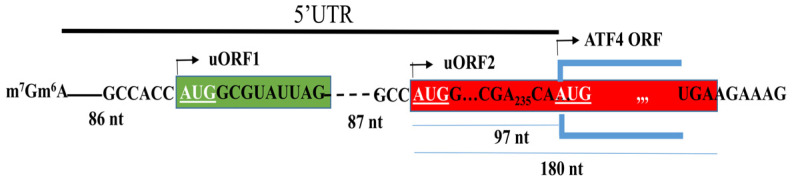
The 5′UTR of human ATF4 mRNA contains two open reading frames (uORFs): uORF1 (green) and uORF2 (red). uORF1 consists of four codons, while uORF2 spans sixty codons, twenty-seven of which overlap with the main ATF4 ORF. The start codons of uORF1, uORF2, and ATF4 ORF are underlined.

The 5′UTR of mammalian ATF4 mRNA contains multiple uORFs, with uORF1 and uORF2 being the best characterized in human ATF4 mRNA [52,56,57]. uORF1 comprises four codons, while uORF2 spans sixty codons, twenty-seven of which overlap with the main ATF4 ORF (Figure 3). A less characterized single-codon uORF consisting of an AUG followed by an immediate stop codon lies downstream of uORF1 [58]. Both uORF1 and uORF2 contain start codons (uAUG1 and 2) with favorable Kozak sequences, promoting efficient recognition by translation initiation complexes.

**Figure 4:**
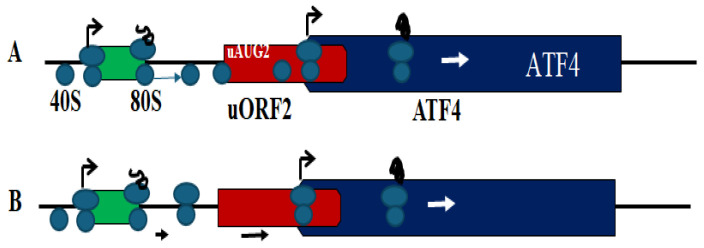
Original (**A**) and updated (**B**) models of the delayed translation reinitiation mechanism of stress-induced ATF4 translation. (**A**) A portion of the migrating 40S ribosomes scans past the initiation codon uAUG2 of uORF2 before the TC can bind, skipping uORF2 translation, while initiating translation at the ATF4 ORF. (**B**) A subset of post-terminating 80S ribosomes remain associated with the mRNA, bypass uORF2, and initiate ATF4 translation. In this model, both uORF2 and ATF4 are translated.

These uORFs control ATF4 translation through a delayed translation reinitiation (REI) mechanism [52,59]. After uORF1 translation, the 60S subunit dissociates, while the 40S ribosome continues scanning downstream. Under non-stress conditions, abundant ternary complexes (TCs) allow rapid TC reassociation, leading most ribosomes to reinitiate at uORF2. Translation of uORF2, which overlaps the ATF4 ORF, is believed to suppress ATF4 expression (Figure 4). The precise inhibitory mechanism remains unclear and requires further investigation.

**Figure 5:**
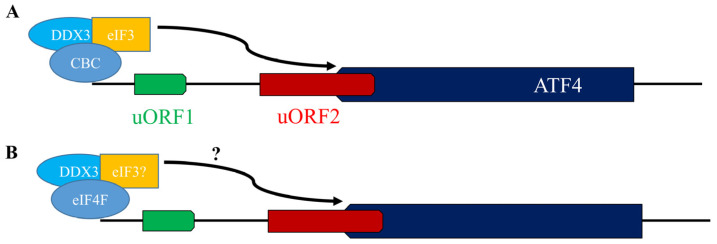
(**A**) ATF4 translation in SAS cells. DDX3, CBC, and eIF3 may form a functional complex to force ribosomes to scan through uORF2 bypass despite the availability of TC, enabling ATF4 translation. (**B**) DDX3-eIF4F-mediated ATF4 translation. Under acute ER stress, DDX3 associates with eIF4F to promote ATF4 translation. How DDX3-containing eIF4F activates ATF4 translation remains to be investigated.

During translational stress, reduced TC availability delays ribosomal reassembly, allowing some ribosomes to bypass the uORF2 start codon and initiate at the main ATF4 ORF (Figure 4A) [52,59]. However, ribosome profiling [1,52] and T-cell tracing peptide studies [52,60] suggest that activation of ATF4 translation may occur without a substantial decrease in uORF2 translation. Moreover, QTI-seq data [52,61] indicate that reinitiation may involve post-terminating 80S rather than 40S ribosomes, challenging the classical REI model and underscoring the complexity of ATF4 translational control (Figure 5B). In the updated REI model, even limited ribosomal escape from uORF2 can markedly enhance ATF4 translation. While uORFs are central to ATF4 translational regulation, as covered in several comprehensive reviews, emerging evidence highlights an equally important role for trans-acting factors, discussed in this review.

### 3.1. Cap-Binding Complexes

Two cap-binding complexes, the nuclear cap binding complexes (CBCs) and cytoplasmic eIF4F have been implicated in regulating ATF4 translation. Activation of ATF4 translation by CBCs was described in oral squamous cell carcinoma (OSCC) cells (SAS line). In these cells, ATF4 translation is abnormally elevated [62]. This activation likely arises from a stress-like environment driven by overexpression of DDX3, a DEAD-box RNA helicase and CBC partner [58,62]. Because ATF4 translation in SAS occurs without external stress, eIF2α phosphorylation is unlikely involved. Similar ATF4 activation has been observed in cells with reduced expression or activity of eIF2B, eIF5, or eIF5B, where decreased TC availability or ribosome-binding capacity drives translation independently of eIF2α phosphorylation. Whether these factors are altered in SAS, contributing to ATF4 translation, remains unknown.

In SAS cells, DDX3 depletion suppresses ATF4 translation, while DDX3 overexpression enhances translation of a *Renilla* luciferase reporter containing the ATF4 5′UTR, confirming that DDX3-dependent translation relies on ATF4 uORFs [62]. Mutation of uAUG1 (uORF1 start codon) has no effect, whereas mutation of uAUG2 (uORF2 start codon) significantly increases translation [62], indicating that DDX3 may help ribosomes bypass uORF2-mediated inhibition, possibly via a mechanism distinct from the classical REI model (Figure 5A).

The requirement for both DDX3 and CBCs suggests a functional DDX3–CBC complex. Although CBCs are typically nuclear [63], overexpressed DDX3 localizes mainly to the cytoplasm in SAS cells [62], possibly recruiting CBCs to cytoplasmic ATF4 mRNA and stabilizing their interaction. The mechanisms by which ribosomes are recruited to or assisted by the DDX3–CBC complex remain unclear. Immunoprecipitation data show that the complex also contains eIF3 and eIF4E [62], representing noncanonical and canonical cap-binding factors, respectively. Unexpectedly, eIF4E appears dispensable for ATF4 translation in SAS [62], unlike in stress-induced translation, where it plays a key role. The role of eIF3 in ATF4 translation is described below (see Section 3.5).

We have found that in hepatocarcinoma cells under acute ER stress (2–4 h treatment with thapsigargin or sorafenib), DDX3 associates with eIF4F to promote ATF4 translation [24] (Figure 5B). Independent studies also found that eIF4F is minimally required for full ATF4 induction in MEFs, mESCs, and HEK293 cells treated with thapsigargin, a classical ER stress inducer [38,64]. eIF4F’s role appears restricted to acute stress, while eIF3 sustains translation under chronic stress as a noncanonical cap-binding complex. This shift aligns with significantly reduced activities of eIF4F and its upstream mTORC1 activating pathway during chronic stress. Under acute stress, however, the eIF4F–mTORC1 axis remains partially active, supporting ATF4 translation [24,64]. Notably, targeting the eIF4F–mTORC1 axis also disrupts SG assembly [65,66,67], which may increase ATF4 mRNA availability for translation and attenuate the impact of eIF4F and mTORC1 inactivation on ATF4 translation. Enforcing SG assembly in cells with inactive eIF4F or mTORC1 could further clarify their effects on ATF4 translation.

### 3.2. eIF2B

**Figure 6:**
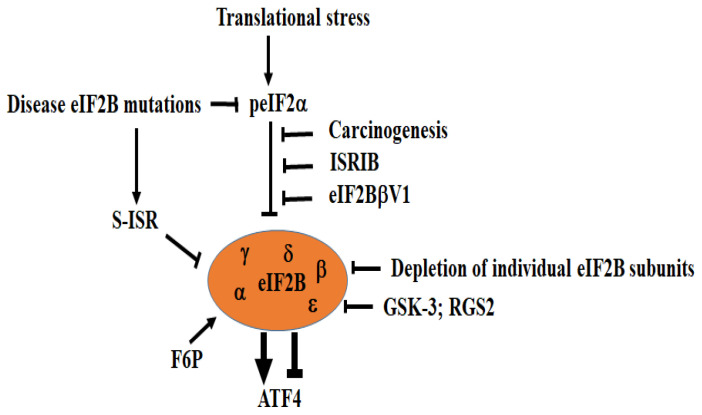
eIF2B is central in regulating ATF4 translation. Under translational stress, the GEF eIF2B is inhibited by peIF2α, activating ATF4 translation. The effects of peIF2α antagonized by either ISRIB or the expression of the eIF2bV1 isoform prevents ATF4 translation. Similarly, cancer cells such as CRC are also resistant to peIF2α, potentially by expressing high levels of eIF2B. Disease eIF2B mutations or depletion of eIF2B individual subunits can induce ATF4 translation. Phosphorylation of eIF2Be by GSK-3 or expression of RGS2 may also induce ATF4 translation. Arrowheads denote activation and blunt-ended lines indicate inhibition.

eIF2B, the main target of peIF2α, is a large GEF complex composed of five subunits (α–ε) [68]. The α, β, and δ subunits form a regulatory sub-complex, while the γ and ε subunits constitute the catalytic core [69]. eIF2 and peIF2 bind eIF2B at distinct sites with different affinity [70,71,72,73,74]. peIF2 binds more tightly, allosterically altering eIF2B conformation, inhibiting its GEF activity [69,70,71,72,73,74,75], lowering TC levels, and inducing ATF4 translation. eIF2B levels are generally lower than eIF2 [76], making TC formation and downstream translation control highly sensitive to changes in peIF2. Proper ATF4 regulation thus requires tight control of eIF2B GEF activity and expression [23] (Figure 6).

Partial loss-of-function mutations in eIF2B genes cause a neurodegenerative disorder called vanishing white matter disease (VWMD), a fatal childhood and adult-onset leukodystrophy [77,78]. VWMD patients are vulnerable to traumatic brain injury and neuroinflammation, which can cause phosphorylation of eIF2α and chronic activation of the ISR [77,79,80,81,82]. VWMD cell lines with endogenous hypomorphic mutations in any eIF2B subunit show reduced GEF activity, impairing GDP–GTP exchange on eIF2 and lowering TC [38,83,84,85,86,87,88], which can aberrantly induce ATF4 translation. This occurs without peIF2α activation and with minimal inhibition of global translation [38]. eIF2B deficiency exacerbates peIF2α under acute ER stress and alters cell recovery from stress, which may explain the sensitivity of VWMD cells (e.g., leukocytes) to chronic ER stress [85]. These findings are consistent with data showing selective damage to astrocytes and oligodendrocytes in VWMD [84,89], suggesting eIF2B mutations trigger toxic metabolic reprogramming in these cells, potentially driven by ATF4. Further studies should explore how eIF2B deficiency-driven ATF4 translation dysregulation contributes to VWMD and whether targeting this translation can rescue disease features. It should be noted, however, that expressing VWMD mutants with reduced eIF2B activity in transformed cells such as HEK293 do not activate ATF4 translation or sensitize them to stress [88]. This unexpected result may be due to the high expression of eIF2B in transformed cells, masking the effects of VWMD mutations, though alternatives may exist.

New insights into eIF2B misregulation-mediated ATF4 translation were recently provided. It was shown that either the expression of hypomorphic eIF2Bε mutants (reducing GEF activity by 40%) in mESCs or the shRNA-mediated eIF2Bε knockdown in MEFs activates a noncanonical ISR termed split ISR (s-ISR), characterized by ATF4 translation [38]. Activation of s-ISR occurs independently of eIF2α phosphorylation [38], and thus cannot be blocked by targeting that phosphorylation. Canonical ISR targets like GADD34 (peIF2α phosphatase), CHOP, or BiP are not induced during s-ISR, making ATF4 a central marker of s-ISR [38]. Activation of the s-ISR partially reduces general translation and activates an ATF4-dependent metabolic gene program that is distinct from the canonical ISR-mediated program, essential for cells’ adaptation to eIF2B deficiency [38]. ATF4 translation that occurs during s-ISR depends on eIF4E and DDX3 [38]. To test uORF involvement, mESCs expressing an ATF4 ΔuORF1 mutant (uORF1 ATG mutated to ATA) were generated via CRISPR-Cas9. These cells did not show ATF4 induction upon eIF2Bε depletion, suggesting s-ISR relies on uORF1 translation [38]. This contrasts with previous luciferase translational data obtained in SAS using the ATF4-FLuc reporter [62]. These SAS data suggested that uORF1 is dispensable for ATF4 translation, possibly due to the high abundance of TC under those conditions, allowing the ribosomes to bypass uORFs and initiate ATF4 translation in a DDX3–CBC–eIF3-dependent manner. In ΔuORF1 mESC cells with eIF2B deficiency, ATF4 mRNA still associates with ribosomes, possibly due to increased uORF2 translation, which inhibits ATF4, though this requires validation with approaches such as ribo-seq. Depletion experiments may also clarify the role of eIF4E and DDX3 in uORF1 translation during s-ISR. As in the case of acute ER stress [24], eIF4E and DDX3 may be part of a DDX3-containing eIF4F complex essential for s-ISR-induced ATF4 translation, though this has not been demonstrated yet. Whether this eIF2B downregulation-mediated s-ISR mechanism is conserved in humans remains to be determined.

eIF2B was recently shown to sense aberrantly high peIF2α in APC tumor suppressor-mutant colon cancer cells (CRCs) [90]. This role of eIF2B is essential to buffer the negative effects of peIF2α, thereby balancing translation required for CRC proliferation. Targeting eIF2B impairs CRC viability by disrupting translation of APC mutation-induced mRNAs. Depleting β, γ, δ, or ε disrupts eIF2B decamer formation in CRC, lowers peIF2α, and reduces translation. A minimal βγδε complex lacking α retains GEF activity, but also lowers peIF2α. Mutations disrupting eIF2Bα dimerization reduce peIF2α, but induce the ISR CHOP marker. Mutating δ, which contacts peIF2α, also reduces peIF2α while preserving eIF2B complex formation. In contrast, depleting or mutating ε, which alters eIF2B GEF activity, has no effect on peIF2α, suggesting decamer formation alone lowers peIF2α. eIF2Bα or δ depletion in CRCs induces ATF4 and downstream canonical ISR genes (CHOP, DDIT3, PPP1R15A and B). Among these ATF4-target genes, PPP1R15A encodes GADD34, which during transient stress is responsible for recruiting PP1C phosphatases that induce dephosphorylation of eIF2α, thereby partially restoring the activity of eIF2B and general translation, turning off ATF4 translation [23]. ATF4-mediated induction of GADD34 may thus account for the reduction of peIF2α in eIF2Bα- or δ-depleted CRC. The induction of ATF4-downstream ISR genes argues against activation of an s-ISR in CRC. Whether upregulation of ATF4 upon eIF2Bα or δ depletion involves eIF4F, DDX3, or other factors is unclear. Nevertheless, these findings indicate that targeting eIF2B components in cancer cells such as CRCs that express high levels of peIF2α can regulate ATF4 translation independently of peIF2α. Implications for this regulation in CRC development remain to be established.

eIF2B is also regulated by phosphorylation, which may impact ATF4 translation. GSK-3 phosphorylates the catalytic ε subunit at Ser-540, inhibiting GEF activity [91,92,93] and increasing eIF2B susceptibility to peIF2α. This is primed by DYRK2 phosphorylation, enhancing GSK-3 repression of eIF2B [94]. Consistent with the potential role of GSK-3 in targeting eIF2B, its activity is increased during stress. These studies using eIF2B mutants were challenged by the finding [95] that GSK-3 inhibitors had no effect or even a positive effect on ATF4 translation under ER stress.

While no known factors regulate the eIF2–eIF2B interaction to control ATF4 translation under stress, the synthetic bisglycolamide ISRIB (ISR inhibitor) was identified and shown to directly bind eIF2B, rendering the GEF largely independent of p-eIF2α control. By antagonizing peIF2α-induced conformational changes in eIF2B and preserving GEF activity, ISRIB restores TC levels, thus downregulating ATF4 [75,86,96,97,98,99,100,101]. This shows eIF2B is druggable and supports developing compounds to restore eIF2B function and ATF4 level in disease. Fructose-6-phosphate (F6P), identified in a screen for binding to eIF2Bα [102], was shown to increase eIF2B GEF activity. The finding that metabolites such as F6P activate eIF2B suggests that changes in cell metabolism may directly enhance eIF2B activity and thus may circumvent its inactivation under stress, affecting translational control of ATF4.

Currently, studies investigating regulation of eIF2B by interactors in ATF4 translation are very limited. Regulator of G protein signaling 2 (RGS2) is upregulated by stress and can augment translational attenuation associated with peIF2α [103]. RGS2 can interact with eIF2Bε, blocking its ability to activate eIF2 for translation, and inhibit de novo protein synthesis [104]. The expression of either full-length RGS2 or its eIF2B-interacting domain (RGS2eb) via lentivirus in NIH3T3 cells significantly increases levels of ATF4, possibly at the translational level [104]. Translational assays and depletion experiments are needed, however, to validate the role of RGS2 in regulating translation of ATF4, defining the underlying mechanisms. It is also likely that devising functional screening experiments for factors that bind eIF2B subunits will help identify regulators of eIF2B in ATF4 translation.

ATF4 translation is also regulated by eIF2Bδ isoforms [105]. Alternative splicing of eIF2Bδ pre-mRNA generates two isoforms, V1 and V2, that have been shown to be differentially expressed in neuroblastoma lines. Expression of V1, but not V2, renders cells insensitive to peIF2α under ER stress. Though peIF2α levels remain intact, ATF4 and its targets are not induced and general translation is maintained, preventing canonical ISR activation. Co-immunoprecipitation suggests V1 disrupts eIF2B–peIF2α interaction, preserving TC levels, though the underlying mechanism remains unknown. Translatomic studies using ribosome- or polysome-seq should help determine if V1 reprograms translation toward alternative ISR pathways. The regulation of eIF2Bδ splicing events and their roles in cell processes like differentiation or transformation via ATF4 translation are currently unknown. Key aspects of the central role of eIF2B and its regulation in ATF4 translation are summarized in Figure 6.

The activity of eIF2B can also be targeted indirectly during stress, affecting ATF4 translation. This involves inhibition of the eIF2α phosphorylation by targeting its kinases with inhibitors such as PERKi, namely GSK2606414 and GSK2656157. PERKi inhibits initial activation of the c-ISR during ER stress and prevents ATF4 translation. These inhibitors are considered new promising therapies for many human ER stress-associated diseases, in particular cancer and neurodegeneration [106,107], potentially involving downregulation of ATF4 translation. However, loss-of-function mutations reported in PERK are associated with the development of Wolcott–Rallison syndrome (WRS), characterized by neonatal diabetes, osteoporosis, digestive dysfunctions, and hepatic complications [108]. The inability of PERK to appropriately induce peIF2α and translational control in WRS leads to inappropriately high protein synthesis and probably lack of ATF4 induction, which together prevent activation of an effective c-ISR for stress recovery. Similarly, loss-of-function mutations in GCN2 have been linked to stress-associated pulmonary disorders [109,110], potentially involving the loss of peIF2α-mediated translational control of ATF4. On the other hand, mutations in PPP1R15B (CReP), which contributes to constitutive dephosphorylation of peIF2α, also leads to diabetes growth defects, bone and kidney deformities, and microcephaly [111], potentially involving pathological hyperactivation of the c-ISR and ATF4 translation due to uncontrolled eIF2α dephosphorylation. In this case, the basal peIF2α levels would be expected to be enhanced in these conditions. Whether eIF2B can sense this aberrant elevated peIF2α due to CReP mutations to buffer its negative effects and balances translation including ATF4 translation required for cell survival is currently unknown. Constitutive eIF2α phosphorylation can also be induced by drugs such as salubrinal, which targets GADD34–PPi and CReP–PPi complex phosphatases, altering their activity in eIF2α dephosphorylation [112]. Salubrinal protects cells from toxic components, potentially involving ATF4 induction independently of eIF2B, and this may have therapeutic applications.

### 3.3. eIF5

eIF5 regulates eIF2 activity by interacting with both its GTP- and GDP-bound forms [76,113,114,115,116,117]. As a GTPase-activating protein (GAP), eIF5 promotes GTP hydrolysis on TC, facilitating its recycling by generating eIF2-GDP. eIF5 also enhances TC recruitment to the PIC [76,113,114,115,116,117]. Depletion of eIF5 increases ATF4 translation in HEK293 cells, consistent with its role in maintaining TC availability [118]. Uncontrolled binding of eIF5 to GDP-bound eIF2 can however hinder eIF2B-mediated nucleotide exchange, reducing TC reformation. Accordingly, overexpressed eIF5 inhibits TC formation and downregulates translation, mimicking the effects of eIF2α phosphorylation. Overexpression of eIF5 in HEK293 and HT1080 cells activates translation of FLuc from the ATF4-Luc reporter, although less effectively than ER stress [119]. A mutant eIF5 unable to bind eIF2 has a reduced effect, suggesting eIF5 promotes ATF4 translation by interfering with eIF2 function [119]. Overexpressed eIF5 also inhibits translation of uORF2 from uORF2-Luc reporter, suggesting a role in promoting ribosomal reinitiation at the ATF4 start codon [119]. Similar effects are observed with overexpression of eIF5-mimic proteins (5MPs) [119], which lack GAP activity, but retain eIF2-binding domains [120]. Expressing 5MPs in MEFs with an eIF2α Ser 51-to-Ala mutation also induces ATF4-FLuc [119], indicating that 5MPs affect eIF2 function independently of phosphorylation of its α subunit. 5MPs may competitively inhibit eIF2 function and TC recycling, thus promoting ATF4 translation. Both eIF5 and 5MPs associate with eIF3 and eIF2B, and their overexpression may disrupt PIC formation or scanning, contributing to modest ATF4-FLuc activation compared to ER stress-induced eIF2α phosphorylation. Whether eIF5 or 5MP overexpression induces endogenous ATF4 translation remains untested, however. Similarly, the physiological role of 5MPs in ATF4 regulation via depletion studies has yet to be established. Nonetheless, these findings highlight the role of eIF5 interaction with eIF2 in modulating ATF4 translation.

### 3.4. eIF5B

**Figure 7:**
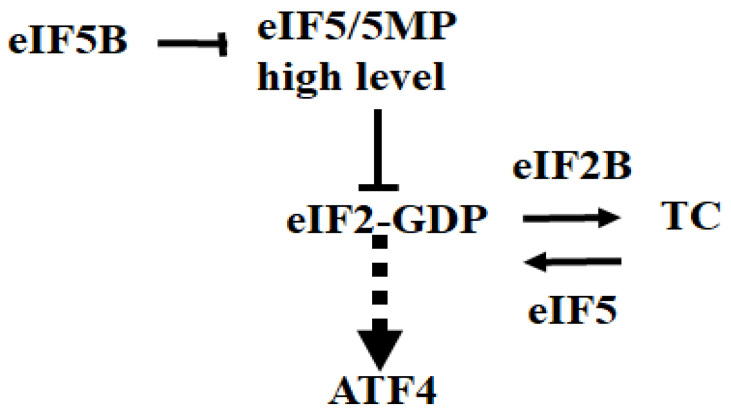
High levels of eIF5/5MPs can trap GDP-bound eIF2, preventing eIF2B-mediated nucleotide exchange, reducing TC reformation, which may trigger ATF4 translation. eIF5B can antagonize the action of free eIF5, and its depletion can activate ATF4 translation. Both eIF2B and eIF5 are required for ATF4 downregulation. Depletion of either eIF2B or eIF5 activates ATF4 translation by downregulating TC. Blunt-ended lines indicate eIF2-GDP trapping. Whether trapping of eIF2-GDP is sufficient to induce ATF4 translation remains to be established, as indicated by the arrowhead with dashed lines.

eIF5B is functionally homologous to bacterial and archaeal IF2, which delivers Met-tRNAi to the ribosomes [121,122,123]. Under normal growth conditions, eIF5B stabilizes Met-tRNAi binding to the ribosome while assisting 40S and 60S joining [124] for efficient cap-dependent translation [125]. In yeast, eIF5B can substitute for eIF2α in delivering Met-tRNAi during translation initiation of uORF-containing mRNAs [126], while in human cells (HEK293 and U2OS), its depletion strongly activates ATF4 translation, as shown by polysome profiling and translation of ATF4-Luc reporters [127]. This derepression depends on an intact uORF2, suggesting that eIF5B promotes initiation at uORF2, similar to conditions where the ternary complex is abundant [127]. Mechanistically, eIF5B may prevent free eIF5 accumulation, which should increase upon depletion. Free eIF5 has the ability to sequester eIF2-GDP and impairs eIF2B-mediated GDP–GTP exchange, thereby limiting TC reformation. eIF5B promotes eIF2-GDP displacement from Met-tRNAi, an essential step for efficient TC recycling. Depletion of eIF5B may thus downregulate TC recycling by slowing down eIF2-GDP displacement from Met-tRNAi. In any case, transcriptomic analysis confirms that eIF5B knockdown induces canonical ISR-related gene expression via ATF4 activation [128]. However, this response differs from that seen with eIF2B depletion in mouse cells, which was shown to activate an s-ISR. Functional interactions between eIF5, eIF5B, and eIF2B in ATF4 translation are summarized in Figure 7.

### 3.5. eIF3

The role of eIF3 in stabilizing mRNA-40S ribosome interactions during translation initiation is well established, but recent evidence shows eIF3 also contributes to reinitiation, notably in uORF-containing transcripts like ATF4 [129,130]. Mammalian eIF3 comprises 12 subunits [131,132], with differential requirements for ATF4 translation under stress. Depletion of eIF3c, d, or g, but not eIF3I reduced induction of ATF4 in MEFs under acute ER stress [64]. Reporter assays using an ATF4-FLuc reporter with intact uORF1, but with uORF2 disrupted to permit stress-independent FLuc translation, were used in HeLa cells. In these assays, depletion of eIF3h, but not eIF3k reduced FLuc levels, supporting a role for eIF3h in reinitiation [129,130]. The data also suggest that eIF3 can promote full ribosome recycling by stabilizing the association of the post-terminating 80S ribosome with the mRNA, possibly recruiting other initiation factors to enable scanning and reinitiation. eIF3 must therefore remain associated with the 80S ribosome during uORF translation [129,130]. Whether eIF3 actively promotes uORF translation elongation and how its interaction with post-terminating ribosomes is regulated remain unknown. In the following sections, we discuss the roles eIF3a and eIF3d in ATF4 translation, which have been studied more.

#### 3.5.1. eIF3a

The role of eIF3a remains unclear due to its broader effect on general translation. Recently, eIF3-seq was used to investigate eIF3a’s role in translation reinitiation [133]. Data showed that in MEFs under acute amino-acid deprivation stress, eIF3a is retained on elongating 80S ribosomes, facilitating reinitiation. This effect is linked to de-O-GlcNAcylation of eIF3a at S225, as inhibition of this modification suppressed ATF4 translation, while introducing an S225 mutation via CRISPR genome editing promoted ATF4 translation even in nutrient-rich conditions [133]. This mutation allowed dissection of reinitiation-specific functions of eIF3a without compromising its essential role in general translation. Reporter assays using ATF4-FLuc confirmed enhanced FLuc translation in S225G mutant cells, dependent on uORF1 integrity, which is necessary for eIF3a interaction with ATF4 mRNA [133]. Though S225G maintained RNA interaction despite uORF1 deletion, whether it rescues translation in ∆uORF1 cells was not tested. These findings indicate that nutrient stress-induced ATF4 translation involves eIF3a de-O-GlcNAcylation, enhancing ribosome retention for reinitiation. Pharmacological disruption of cellular O-GlcNAcylation by treatment with urolithin A, a mitochondrial stress inducer [134], significantly elevates ATF4 expression in various human cell lines [13], further supporting that de-O-GlcNAcylation promotes ATF4 translation. Whether de-O-GlcNAcylation of eIFs such as eIF4G or ribosomal proteins [135,136,137] similarly impacts their function in ATF4 translation is currently unknown. A fraction of eIF3a, along with O-GlcNAcylated factors such as eIF4G and ribosomal proteins, accumulates in SGs under stress [135,136,137]. Whether this eIF3a-associated SG fraction represents an inactive O-GlcNAcylated form, and whether the de-O-GlcNAcylated eIF3a form escapes SG association to activate ATF4 translation are possibilities that may be addressed to understand the regulation of eIF3 subunits partitioning between SGs and translational machinery, required for ATF4 translation.

#### 3.5.2. eIF3d

**Figure 8:**
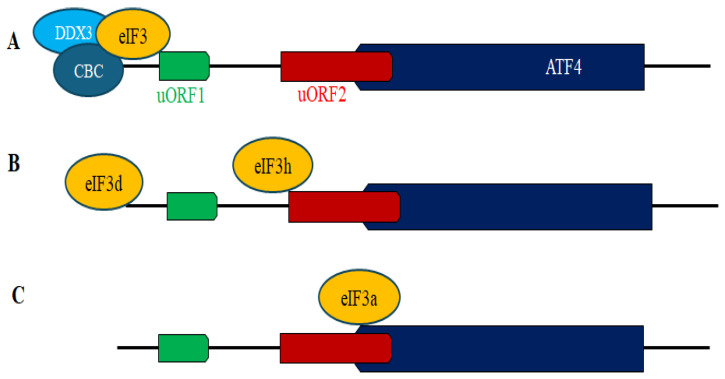
Activation of ATF4 translation via eIF3. (**A**) eIF3 acts as a component of the DDX3–CBC complex in SAS. (**B**) eIF3d acts as a non canonical cap-binding protein in HEK293 cells under chronic ER stress. Unphosphorylated eIF3d may also act similarly under chronic glucose deprivation conditions. In HeLa cells, eIF3h stabilizes the association of post-terminating 80S ribosomes with the mRNA. (**C**) eIF3a is de-O-GlcNAcylated in amino acids-deprived MEFs.

Mammalian eIF3d functions as a noncanonical cap-binding protein that promotes translation of uORF-containing mRNAs [138], including ATF4 [64,139]. While eIF3d collaborates with eIF4F during acute ER stress, it sustains ATF4 translation under chronic stress to activate a chronic ISR program independently of eIF4F [64,139]. Hman eIF3d can make specific contact with the cap: cap-binding mutants disrupt 48S complex assembly despite intact RNA binding [138], highlighting eIF3d’s role in an alternative cap-dependent translation mechanism, although this was not directly shown for ATF4. eIF3d is phosphorylated at S528/S529, near its cap-binding domain, modifications lost during prolonged (48 h) glucose starvation [140]. Expression of phosphomimetic mutants in HEK293 cells blocks eIF3d cap binding, while phosphoinhibitory mutants restore cap interaction and translation of target mRNAs, demonstrating that eIF3d phosphorylation represses cap binding in non-stressed cells, while dephosphorylation activates a stress-adaptive translation program [140]. This was demonstrated using subunit-seq, a method isolating the cap-binding domain of eIF3d for RNA profiling. Briefly, an eIF3d protein with an internal tobacco etch virus (TEV) cleavage site proximal to the cap-binding motif and C-terminal hemagglutinin (HA) affinity tag (eIF3d^TEV^) was engineered and expressed in HEK293 cells [140,141]. HA-tagged eIF3d complexes were isolated and subjected to TEV protease cleavage to specifically isolate eIF3d cap-binding domain–RNA complexes. Bound RNA was subjected to deep sequencing, identifying hundreds of mRNAs translated via direct cap-binding activity of eIF3d during glucose deprivation, enriched in metabolic functions. Surprisingly, ATF4 mRNA was not among these, suggesting eIF3d regulates its translation independently of direct cap binding in this context. The role of eIF3 in ATF4 translation is summarized in Figure 8.

Using subunit-seq during chronic ER stress (induced by prolonged treatment with thapsigargin), the identified eIF3d-bound mRNAs differed from those during glucose deprivation, suggesting stress-specific regulation of eIF3d cap binding [139]. In HEK293 cells with phosphomimetic eIF3d, cap binding and adaptation to chronic stress were impaired [139]. These cells failed to sustain eIF2α phosphorylation or ATF4 expression during chronic stress despite responding normally during the acute phase, indicating that eIF3d dephosphorylation is required to maintain the ISR during prolonged stress.

Among the eIF3d-bound mRNAs identified during chronic ER stress were GCN2 and ALKBH5, whose translation activation was confirmed by polysome profiling [139]. GCN2, dispensable during acute ER stress (dominated by PERK), contributes significantly to ATF4 induction in the chronic phase, presumably by sustaining eIF2α phosphorylation [139]. ALKBH5 encodes an RNA demethylase targeting N^6^-methyladenosines (m^6^A) and N^6^,2′-O-dimethyladenosine (m^6^Am) marks. m^6^A-eCLIP experiments [139] showed that ALKBH5 expression, mediated by the non-phosphorylated eIF3d mutant promotes demethylation of chronic stress-responsive m^6^A sites, including three in the ATF4 mRNA [139]. Among these, one m^6^Am site lies at the transcription start site adjacent to the cap, and is modified in a cap-dependent manner [139,142]. While its demethylation by ALKBH5 during chronic stress is hypothesized to enhance ATF4 translation, this remains unproven. A conserved m^6^A site (A235 in human, A225 in mouse) in the 5′UTR is demethylated during acute nutrient and ER stresses, and this demethylation is required for ATF4 translation [61,143]. Thus, distinct ATF4 demethylation events occur during acute and chronic stress, mediated by the ALKBH5–eIF3d axis in the chronic phase. How ALKBH5 supports ATF4 translation under prolonged stress remains to be clarified. Overall, these findings establish that eIF3d, through its cap-binding activity and regulated phosphorylation, coordinates a noncanonical cap-dependent translation of stress-regulated mRNAs such as GCN2 and ALKBH5 during chronic stress, contributing to sustained ATF4 translation and translational adaptation. These findings further illustrate the complexity of the translational mechanisms acting as a switch under stress, converging to the regulation of ATF4 translation. Identifying kinases and phosphatases that regulate the activity of eIF3d in ATF4 translation should bring new insights linking specific signaling pathways to the control of specialized translation under stress. The role of ALKBH5 in the translation of ATF4 mRNA, particularly during acute stress, is further detailed below.

### 3.6. Non-Canonical (eIF2D, DENR, and MCTS1) eIFs

eIF2D, DENR (density-regulated reinitiation and release factor), and MCTS1 (multiple copies in T-cell lymphomas 1) are noncanonical eIFs that can deliver Met-tRNAi^Met^ to ribosomes [5,144,145,146]. DENR and MCTS1 form a functional heteromeric complex while eIF2D is a monomeric protein that contains the functional domains of both DENR and MCTS1 combined into one protein. The N-terminal part of eIF2D is homologous to MCTS1, and its C-terminal is homologous to DENR. eIF2D and DENR have been shown to be redundantly required for ATF4 expression during development and under stress [5] using genetic studies in *Drosophila* and CRISPR-Cas9 editing and depletion experiments in human HAP1 (a near-haploid human cell line) cells. Data showing that targeting either eIF2D or DENR modestly affects the steady-state level of ATF4 mRNA support both eIF2D and DENR promoting ATF4 expression in flies and HAP cells, mainly at the translational level. Ribosome footprints and RNA-seq data showed that DENR deficiency in HeLa (DENR^KO^, generated using crisper-cas9), reduced ribosome occupancy at the ATF4 ORF, further supporting its translational role [147]. DENR^KO^ cells have also reduced ribosome footprint density on uORF2, but showed unchanged footprint density on uORF1, suggesting a role of DENR in translation of both uORF2 and ATF4. DENR^KO^ cells have normal ATF4 RNA levels, but reduced ATF4 protein levels and reduced FLuc expression of the ATF4-Luc reporter, consistent with impaired ATF4 translation. MCTS1^KO^ HeLa cells have also reduced ATF4 protein levels, suggesting a similar translational role of MCTS. DENR^KO^ cells still express eIF2D, but not MCTS1, as DENR and MCTS1 stabilize each other [148]. Knockdown of eIF2D in the DENR^KO^ cells further prevented the stress-inducibility of ATF4. Similar results have been observed in HT1080 fibrosarcoma cells, indicating a broader role of these proteins in ATF4 translation. The contribution of eIF2D and possibly of DENR and MCTS1 to ATF4 translation varies between cell lines, however, likely due to differences in their activity or expression. Nevertheless, these findings suggest that eIF2D and its homologs may constitute alternatives factors that deliver the initiator tRNA to ribosomes under conditions of eIF2α inactivation to sustain ATF4 translation, though this remains to be demonstrated.

### 3.7. RNA-Modifying Enzymes

#### 3.7.1. PUS7

PUS7 is a member of the pseudouridine synthase family that catalyzes RNA pseudouridylation (Ψ), a common RNA modification that increases RNA stability and translation [149,150,151,152]. The role of PUS in regulating Ψ-mediated ATF4 translation was recently investigated in neuroblastoma cells [153]. Expression studies showed that PUS7 is highly abundant in MYCN-amplified neuroblastoma cell lines compared to non-MYCN-amplified cells. Elevated expression of PUS7 in MYCN-amplified neuroblastoma cell lines mediates translation of ATF4 mRNA, possibly accounting for its high basal abundance. PUS7 is also required for the induction of ATF4 translation in non-MYCN-amplified neuroblastoma cell lines upon ER or amino-acid deprivation stress. The catalytically inactive mutant PUS7 D294A, in which the aspartate residue at the position 294 is replaced by alanine, cannot support ATF4 translation, indicating that PUS7-mediated ATF4 translation involves its Ψ activity, which is further confirmed using PUS7 inhibitors.

Quantitative profiling of PUS7-dependent Ψ sites in poly(A) RNAs from the MYCN-amplified neuroblastoma BE(2)-C cell line by nanopore sequencing identified MCTS1 as a PUS7 target for Ψ. PUS7 knockdown significantly reduced Ψ levels at the identified sites, indicating their PUS7-mediated pseudouridylation. PUS7 depletion-mediated loss of Ψ in MCTS1 mRNA had no effect on its level, but significantly reduced the level of its association with translating ribosomes, downregulating MCTS1 level in BE(2)-C cells. These data suggest that PUS7-mediated pseudouridylation of MCTS1 mRNA promotes its translation by inducing preferential association with ribosomes. Because depletion of MCTS1 phenocopied the effect of PUS7 knockdown in the suppression of ATF4 induction by amino-acid deprivation or ER stress in BE(2)-C cells, the authors concluded that MCTS1 is a key downstream PUS7 target for efficient ATF4 translation. Whether PUS7 also targets eIF2D, the MCTS1 homolog, for ATF4 translation was not investigated. Experiments using MCTS1-constituted Ψ mutants that are resistant to PUS7 knockdown would be helpful to validate the role of the PUS7–MCTS1 axis in ATF4 translation.

Mutational studies suggest that the integrity of the one-amino-acid uORF of ATF4 mRNA, which lies upstream of uORF1, is required for PUS7–MCTS1 axis-mediated ATF4 translation [153], though this remains to be confirmed. Nanopore RNA-sequencing data from control and PUS7 knockdown BE(2)-C cells treated with tunicamycin revealed that PUS7 knockdown also decreased Ψ levels at multiple sites in the ATF4 mRNA. Human cells express two ATF4 mRNA isoforms, V1 and V2, that encode the same protein, but differ in their 5′UTR, with V1 containing an internal segment that does not present in the 5′UTR of V2. V1 and V2 mRNAs have the same 3′UTR targeted by PUS7 [153]. Among Ψ sites affected by PUS7 depletion, U1984 in the 3′UTR of V1, corresponding to U1394 in V2, matches the Ψ site of the consensus PUS7 target sequence “UGUAG” (underlined U indicates the Ψ site). Immunopurified PUS7 significantly pseudouridylated an in vitro transcribed 3′UTR ATF4 RNA oligo at U1984/U1384 in vitro, suggesting that these sites are targets of PUS7-mediated pseudouridylation [153]. Expressing an ATF4 gene V1 with a T1984 to C mutation or V2 with T1384 to C in 293T-ATF4 KO cells showed that eliminating the Ψ site delayed the induction of ATF4 by amino-acid deprivation or ER stress. These data suggest that PUS-mediated Ψ of the 3′UTR of ATF4 mRNA is essential for timely induction of ATF4 by stress. Whether PUS-mediated Ψ of ATF4 mRNA affects ATF4 levels by regulating its translation remains to be demonstrated. Because this Ψ occurs at the 3′UTR, it may not affect the initiation step of translation, which largely involves the 5′UTR. Rather, it may regulate the elongation or termination steps of ATF4 translation, two understudied ATF4 translation regulatory steps. However, the 5′UTR of both V1 and V2 ATF4 mRNA contains a sequence that also matches the PUS7 consensus Ψ site (UGUAG) lying within the one-amino-acid uORF (AUGUAG), suggesting a possible role of PUS7 in regulating translation initiation of ATF4 mRNA via pseudouridylation of the 5′UTR.

Like PUS7, dyskerin pseudouridine synthase 1 (DKC1) is highly expressed in neuroblastoma and is essential for the tumorigenic growth of neuroblastoma cell lines [154]. DKC1 sustains both the basal and stress-induced expression of ATF4 [154]. This function of DKC1 is mediated through pseudouridylation of 28S rRNA, which promotes hnRNP A1 protein expression, inducing internal ribosome entry site (IRES)–dependent translation of the variant V1 ATF4 mRNA [154]. Collectively, these data indicate a critical role of pseudouridine synthases in driving ATF4 translation, particularly in MYCN-overexpressing cancer cells.

#### 3.7.2. RNA Demethylases

Ribosome profiling combined with QTI-seq in MEFs revealed that ATF4 ORF ribosome occupancy increases under acute amino-acid starvation [1,52,61]. This increase occurs without a corresponding reduction at uORF2, as confirmed by reporter assays. Although uORF2 translation was not directly measured, these findings challenged the model in which ribosomes quantitatively bypass uORF2 due to TC unavailability, enabling reinitiation at the ATF4 ORF. The absence of 40S ribosomes at the ATF4 start codon even after starvation, as shown by QTI-seq, aligns with in vitro data [60] suggesting that reinitiation involves post-termination 80S ribosomes migrating from uORF1, rather than dissociated 40S subunits. Whether these migrating 80S ribosomes retain TC or reacquire it remains unclear, but the data support the existence of alternative ATF4 reinitiation mechanisms.

Mass spectrometry identified the RNA demethylase ALKBH5, a protein associating with ATF4 mRNA in MEFs upon acute amino-acid deprivation [61]. ALKBH5 depletion suppresses ATF4 translation independently of eIF2α phosphorylation. Methylation assays showed ALKBH5 targets m^6^A225 at uORF2, while reporter assays using an unmethylated A225G mutant showed increased translation under amino-acid starvation, but not in normal conditions. These data indicate that ALKBH5-mediated demethylation of m^6^A225 at uORF2 of ATF4 mRNA is stress-dependent. Under non-stress conditions, m^6^A225 may promote uAUG2 selection by slowing scanning ribosomes, thereby sustaining uORF2 translation and preventing ATF4 translation. This may occur via m^6^A readers such as eIF3a, which could stall ribosomes at uORF2 [155], or through structural changes or recruitment of RNA-binding proteins that slow scanning. These mechanisms are consistent with m^6^A impeding translation by delaying ribosome movement [156,157]. Under stress, demethylation of m^6^A225 may disrupt reader binding or allow helicases to unwind RNA structures, enabling a subset of ribosomes to bypass uORF2 and initiate ATF4 translation. Further studies are required to determine how ALKBH5-mediated demethylation at A225 affects uORF2 translation and promotes ATF4 translation under acute nutrient stress.

Like ALKBH5, FTO acts as an RNA demethylase [158]. Depletion of the RNA demethylase FTO also reduces ATF4 expression and translation of ATF4 reporter mRNAs in MEFs under acute amino-acid deprivation stress, consistent with in vivo findings that FTO overexpression lowers 5′ methylation and enhances ATF4 translation in both normal and starved mice [61]. How RNA demethylases are targeted to ATF4 mRNA’s 5′UTR under stress and whether they activate ATF4 translation in human cells during acute stress remains unknown. Previous studies identified m^6^A235 as the primary m^6^A site in the 5′UTR of human ATF4 mRNA through m^6^A sequencing (m^6^A-Seq) experiments [159,160,161], and its methylation was validated using the T3 ligase assay [162]. This site is conserved across species [143], aligning with the functionally equivalent m^6^A225 site in mice [61], which supports a conserved mechanism of m^6^A demethylation in ATF4 translation. Using si/shRNA knockdown and reporter assays with ATF4-Fluc, we found that both ALKBH5 and FTO are required for ATF4 translation in human Hep3B cells upon acute ER stress (2 h thapsigargin or sorafenib) [143]. Polysome profiling revealed that both demethylases are required for ATF4 mRNA association with actively translating ribosomes and co-sediment with them [143].

**Figure 9:**
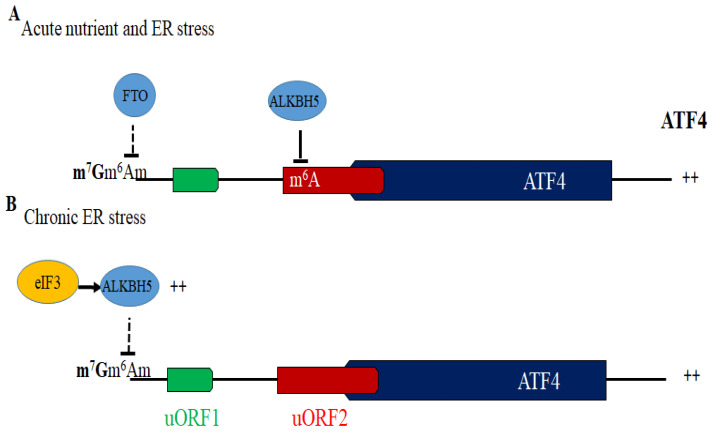
Activation of ATF4 translation via demethylation. (**A**) Both FTO and ALKBH5 are required for the activation of ATF4 translation in MEFs and hepatocarcinoma cells under acute nutrient and ER stress, respectively. ALKBH5 promotes translation of ATF4 mRNA by demethylating its m^6^A225 235 site located at the uORF2. FTO may demethylate the m^6^Am site located adjacent to the cap, though this has not been demonstrated yet. (**B**) In HEK293 under chronic ER stress, translation of ALKBH5 is increased via eIF3. This allows ALKBH5 to sustain translation of ATF4 mRNA via demethylation, potentially at the m^6^Am site located adjacent to the cap. Whether FTO is similarly involved in sustaining translation of ATF4 under chronic stress is not known. ++: Translation activation. Arrowheads indicate activation. Blunt-ended and dashed lines denote inhibition.

In vitro methylation assays demonstrated that ALKBH5 knockdown restored m^6^A levels on a biotinylated human 5′UTR ATF4 RNA reporter, confirming its demethylase activity on A235. FTO knockdown had no effect on A235 demethylation, but impaired thapsigargin-induced translation from an A235G-mutated 5′UTR ATF4-FLuc reporter [143], indicating FTO promotes stress-induced ATF4 translation through a distinct mechanism. FTO preferentially targets cap-proximal m^6^A and m^6^Am and lesser internal m^6^A [163,164]. The ATF4 5′UTR is methylated at the first transcribed adenosine via m^6^Am [139,142], a cap-dependent and FTO-sensitive modification [163,164]. While m^6^Am may regulate mRNA stability or translation [165], its specific role in ATF4 expression remains untested. Reporter assays using m^7^G-m^6^Am or m^7^G-Gm capped FLuc mRNAs could clarify whether m^6^Am represses ATF4 translation and if FTO relieves that repression. Additional methylation assays may validate FTO’s role in demethylating m^6^Am on capped ATF4 5′UTR transcripts. FTO might also act on uncharacterized m^6^A sites predicted within uORF2 [162] independently of m^6^Am. FTO was shown to affect m^6^A levels on ATF4 mRNA in mouse retinal pigmented epithelium cells [166] and in colorectal cancer cells under glutaminolysis inhibition [167], though whether it promotes ATF4 translation via 5′UTR demethylation in these contexts remains unclear. Overall, both ALKBH5 and FTO act as positive regulators of ATF4 translation through demethylation of its 5′UTR (Figure 9).

Both ALKBH5 and FTO are subject to post-translational modifications, including SUMOylation [168,169,170], phosphorylation [169,171,172], lysine acylation [173], and O-GlcNAcylation [174], that may regulate their demethylase activity, but whether these modifications affect association with ATF4 mRNA for demethylation and translation remains unknown. Given the weak RNA-binding capacity of ALKBH5 and FTO [175,176], their recruitment to ATF4 mRNA likely requires co-factors. Proteomic studies are needed to identify partners that direct ALKBH5 and FTO to ATF4 mRNA under stress by regulating their localization.

**Figure 10:**
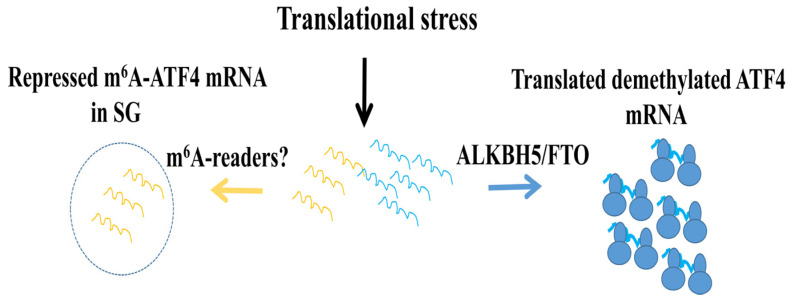
Does translational stress induce partitioning of ATF4 mRNA between SGs and translating ribosomes, regulated by m^6^A modification? In this proposed model, the m^6^A-modified fraction of ATF4 mRNA (yellow) is recognized by m^6^A reader proteins and sequestered into SGs, where it remains untranslated. Conversely, demethylation of ATF4 mRNA by ALKBH5 and FTO enables the demethylated transcripts (blue) to avoid SG association and remain available for translation.

The localization of both ALKBH5 and FTO under stress is intriguing. Although largely nuclear, a fraction of those proteins is cytoplasmic under stress [143,177,178,179]. Our finding that the cytoplasmic ALKBH5 and FTO fractions associate with translating ribosomes [143] is consistent with their role in activating ATF4 translation. While proteins involved in RNA metabolism, including m^6^A writers and readers, have fractions associating with SGs, neither ALKBH5 nor FTO localize in SGs [143,177,178,179]. Whether interactions with ALKBH5 and FTO are required to antagonize localization in SGs, allowing association with ribosomes for demethylation and translation of ATF4 mRNA, remains to be addressed. The finding that both ALKBH5 and FTO are excluded from SGs also raises the possibility that they may regulate the partitioning of ATF4 mRNA between SGs and ribosomes via demethylation. We and others have reported partitioning of ATF4 mRNA between SGs and translating ribosomes under acute ER stress. We found that 25% of ATF4 mRNA associates with SGs, and suggested that this association buffers translation of ATF4, a hypothesis supported by a recent study [48]. In this study, it was shown that disrupting the association of ATF4 mRNA with the ribosomes by translation initiation inhibitors promotes accumulation in SGs induced in U2OS by arsenite treatment. This indicates that the association of ATF4 mRNA with the ribosome limits its recruitment to SGs. Altering uORF sequences in the reporter ATF4 mRNA that are required for its translation also promotes localization in SGs, suggesting that uORFs antagonize SG association in a ribosome-dependent manner. uORFs-mediated translation of ATF4 mRNA relies on demethylations, which may thus be involved in regulating association with SGs [48]. In this case, the partitioning of ATF4 mRNA between SGs and ribosomes may reflect differential m^6^A modification at its 5′UTR. Although experimental evidence supporting that hypothesis is still lacking, several data indicate that m^6^A-modified mRNA differentially partition in and out of SGs under stress. This was first shown in immunofluorescence data generated by us [180] and others [181] showing m^6^A signals enriched within SGs. Importantly, transcriptomic studies showed that m^6^A-modified mRNAs selectively localize with SGs, while mRNAs with low-m^6^A levels are mostly absent from SGs, potentially escaping repression [177]. These findings suggest that m^6^A-driven sorting into SGs may affect translation, and that demethylation may allow specific mRNAs to escape SGs for translation. We speculate that while the m^6^A-modified ATF4 mRNA pool associates with SGs in a repressed from, the demethylated ATF4 mRNA pool escapes SG association for translation (Figure 10). The finding that canonical m^6^A readers, namely YTHDFs, associate with SGs [182] suggests that they may play a significant role in the partitioning of ATF4 mRNAs by driving m^6^A-modified ATF4 mRNA association with SGs. Whether YTHDFs regulate ATF4 mRNA translation under stress remains unknown, however. Noncanonical m^6^A readers such as FXR1 and FMRP may constitute alternatives. Clearly, future studies are required to define the role m^6^A modifications and regulators in SG association and to validate the potential role of this association in keeping a fraction of the m^6^A-modified ATF4 mRNA pool silent. SGs are reversible in nature, as they form under acute stress and can disassemble under prolonged stress. One can assume that under acute stress, SGs may serve as a reservoir of m^6^A-modified ATF4 mRNA, which is then released under prolonged stress for demethylation and sustained expression.

## 4. Conclusions

**Figure 11:**
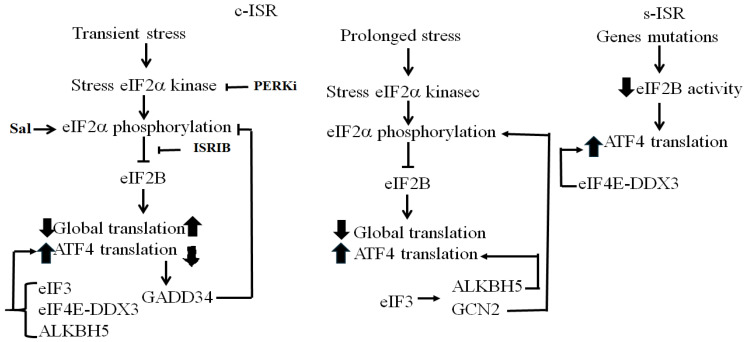
A summary of pathways that activate translation of ATF4 during c-ISR and s-ISR. See text for details. Arrowheads denote activation, while blunt-ended lines indicate inhibition, ⬆ indicates upregulation, ⬇ indicates downregulation.

ATF4 translation was deemed to occur exclusively under translational stress that inhibits global translation initiation via eIF2α phosphorylation. Recent studies showed however that ATF4 translation occurs both in a peIF2α-dependent, activating an c-ISR, and independent manner, triggering an s-ISR (Figure 11). In both cases, downregulation of TC is generally a driving force of that translation, which also requires the activity of canonical eIFs. Among eIFs, eIF2B is unique, as its inactivation is sufficient to drive ATF4 translation during the s-ISR (Figure 11). This ATF4 translation occurs without inducing peIF2α or significantly inhibiting general translation, decoupling ATF4 translation from both processes. Whether under these peIF2α-independent conditions the translation of ATF4 mRNA involves its modification via the activity of RNA demethylases and pseudouridinylation synthases remain to be explored. Both peIF2α-dependent and independent stress conditions can induce SGs, which regulate the partitioning of ATF4 mRNA potentially by house untranslated m^6^A-modified ATF4 mRNA. The partitioning of ATF4 mRNA between SGs and ribosomes may also involve modification of its pseudouridinylation, which is required for efficient translation. Future investigations are required to define the role of RNA modifications, if any, in the spatial regulation of ATF4 mRNA, as a critical regulatory step of translation. The finding that demethylation and pseudouridinylation are critical for ATF4 mRNA translation induced by extrinsic stress raises the possibility that they may also contribute to ATF4 translation induced by intrinsic stress that is generated during physiological cellular processes such as embryonic stem cell pluripotency maintenance [183], immune cell activation [184,185,186], erythroid cell differentiation [187], cell adhesion [188], and cellular senescence [28]. Investigating the contribution of demethylation and pseudouridinylation in ATF4 translation induced by intrinsic stress may also reveal functions of RNA demethylases and pseudouridinylation synthases in controlling important cellular processes via RNA editing. Such RNA editing–based mechanisms may also be responsible for the high translation rate of ATF4 observed in many cancers, warranting investigations.

## Data Availability

No new data were created or analyzed in this study.
